# Supplementary material for: Heat adaptation of phage T7 under an extended genetic code
Source: Virus Evol. 2021 Dec 1;7(2):veab100. doi: 10.1093/ve/veab100 (PMC8923235; doi:10.1093/ve/veab100)
Supplement: veab100_Supp [file veab100_supp.zip › Sequencing_Analysis.zip!/Sequencing_Analysis/A2/evidence/JC_SIDE_1_56.html]

BRESEQ :: Evidence


| New junction evidence | | | | | | | | | | | |
| --- | --- | --- | --- | --- | --- | --- | --- | --- | --- | --- | --- |
|  | | seq id | position | reads (cov) | reads (cov) | score | skew | freq | annotation | gene | product |
| \* | ? | NC\_001604.1 | = 27753 | 287 (0.670) | 183 (0.420) +CGCTATATCTGG | 105/544 | 1.5 | 38.0% | intergenic (–/–) | *–/–* | –/– |
|  | ? | NC\_001604.1 | 27754 = | 318 (0.700) |  |  |  |  | intergenic (–/–) | *–/–* | –/– |

Only 100 of 514 total aligned reads displayed.  

`TCTAGCTATCGGTGGTAACTGCGGGGACCAGTGCTGGTTCGTTACGAGCGACCAAGTGTGGCGACTTAGTGGAAAGGCTAAGCGAAAGTTCCGTAAGTTAATCATGGAGTATCGCGATAAGATGCTTGAGAAGTATGATACTCTTTGGAATTACGTATGGGTAGGCAATACGTCCCACATTCGTTTCCTCAAGACTATCGGTGCGGTATTCCATGAAGAGTACACACGAGATGGTCAATTTCAGTTATTTACAATCACGAAAGGAGGATAACCATATGTGTTGGGCAGCCGCAATACCTATCGCTATATCTGGCGCTCAGGCTATCAGTGGTCAGAACGCTCAGGCCAAAATGATTGCCGCTCAGACCGCTGCTGGTCGTCGTCAAGCTATGGAAATCATGAGGCAGACGAACATCCAGAATGCTGACCTATCGTTGCAAGCTCGAAGTAAACTTGAGGAAGCGTCCGCCGAGTTGACCTCACAGAACATGCAGAAGGTCCAAGCTATTGGGTCTATCCGAGCGGCTATCGGAGAGAGTATGCTTGAAGGTTCCTCAATGGACCGCATTAAGCGAGTCACAGAAGGACAGTTCATTCGGG  >  NC_001604.1/27453‑28052`  

                                                                                                                                                                                                                                                                                                            `|`                                                                                                                                                                                                                                                                                                             
`gctcggagatgtgtataagagacaggACCAGTGCTGGTTCGTTACGAGCGACCAAGTGTGGCGACTTAGTGGAAAGGCTAAGCGAAAGTTCCGTAAGTTAATCATGGAGTATCGCGATAAGATGCTTGAGAAGTATGATACTCTTTGGAATTACGTATGGGTAGGCAATACGTCCCACATTCGTTTCCTCAAGACTATCTGTTCGGTATTCCATGAAGAGTACACACAAGATGGTCAATTTCAGTTATTTACAATCACGAAAGGAGGATAACCATATGTGTTTGGCAGCCGCAATACCTAt                                                                                                                                                                                                                                                                                                             <  1:497470/277‑1 (MQ=255)`  
       `aTCGGTGGTAACTGCGGGGACCAGTGCTGGTTCGTTACGAGCGACCAAGTGTGGCGACTTAGTGGAAAGGCTAAGCGAAAGTTCCGTAAGTTAATCATGGAGTATCGCGATAAGATGCTTGAGAAGTATGATACTCTTTTGAATTACGTATGGGTAGGCAATACGTCCCACATTCGTTTCCTCAAGACTATCGGTGCGGTATTCCATGAAGAGTACACACGAGATGGTCAATTTCAGTTATTTACAATCACGAAAGGAGGATAACCATATGTGTTGGGCAGCCGCAATACCTATcgctata                                                                                                                                                                                                                                                                                                      >  1:392616‑M1/1‑294 (MQ=255)`  
         `cGGTGGTAACTGCGGGGACCAGTGCTGGTTCGTTACGAGCGACCAAGTGTGGCGACTTAGTGGAAAGGCTAAGCGAAAGTTCCGTAAGTTAATCATGGAGTATCGCGATAAGATGCTTGAGAAGTATGATACTCTTTGGAATTACGTATGGGTAGGCAATACGTCCCACATTCGTTTCCTCAAGACTATCGGTGCGGTATTACATGAAGAGTACACACGAGATGGTCAATTTCAGTTATTTACAATCACGAAAGGAGGATAACCATATGTGTTGGGCAGCCGAAATACCTATcgctatatc                                                                                                                                                                                                                                                                                                    >  1:489827‑M1/1‑292 (MQ=255)`  
            `tgggctcggagatgtgtataagagacaggttactagcgaccaagtgtggcgacttagTGGAAAGGCTAAGCGAAAGTTCCGTAAGTTAATCATGGAGTATCGCGATAAGATGCTTGAGAAGTATTATACTCTTTGGAATTACGTATGGGTAGGCAATACGTCCCACATTCGTTTCCTCAAGACTATCGGTGCGGTATTCCATTAAGAGTACACACGAGATGGTCAATTTCAGTTATTTACAATCACGAAAGGAGGATAACCATATGTGTTTGGCATCCGCAATACCTATcgctatatctgg                                                                                                                                                                                                                                                                                                 <  1:589458‑M1/273‑13 (MQ=255)`  
                   `ttaagatccaggtgctggttcctTATCAGTGCACAAGTGTGGCTACTTATTTGAAAGGCGAAGAGAAAGTACCGTAAGTTAATCCTTGAGTATCGCGATAAGATGCTTAATAACTATTAAACACTTTGGAATTACGTATTGGTAGGCAATACGTCCCACATTCGTTTCCTCAATACTATCGGTGCAGTATTCCATGAAGAGTTCACACGAGATGTTCAATTTCAGTTATTTACAATCACGAAAGGAGGATAACCATATGTGTTGGGCAGCCGCAATACCTATcgctatatctggcactata                                                                                                                                                                                                                                                                                          <  1:100160‑M1/290‑20 (MQ=255)`  
                    `gCGGGGACCAGTGCTGGTTCGTTACGAGCGACCAAGTGTGGCGACTTAGTGGAAAGGCTAAGCGAAAGTTCCGTAAGTTAATCATGGAGTATCGCGATAAGATGCTTGAGAAGTATGATACTCTTTGGAATTACGTATGGGTAGGCAATACGTCCCACATTCGTTTCCTCAAGACTATCGGTGCGGTATTCCATGAAGAGTACACACGAGATGGTCAATTTCAGTTATTTACAATCACGAAAGGAGGATAACCATATGTGTTGGGCAGCCGCAATACCTATcgctatatctggcgctatat                                                                                                                                                                                                                                                                                         <  1:569115‑M1/301‑21 (MQ=255)`  
                    `gCGGGGACCAGTGCTGGTTCGTTACGAGCGACCAAGTGTGGCGACTTAGTGGAAAGGCTAAGCGAAAGTTCCGTAAGTTAATCATGGAGTATCGCGATAAGATGCTTGAGAAGTATGATACTCTTTGGAATTACGTATGGGTAGGCAATACGTCCCACATTCGTTTCCTCAAGACTATCGGTGCGGTATTCCATGAAGAGTACACACGAGATGGTCAATTTCAGTTATTTACAATAACGAAAGGAGGATAACCATATGTGTTGGGCAGCCGCAATACCTATcctgtctcttatacacatct                                                                                                                                                                                                                                                                                         >  1:299454‑M1/1‑281 (MQ=255)`  
                         `gACCAGTGCTGGTTCGTTACGAGCGACCGAGTGTGGCGACTTAGTGGAAAGGCTAAGCGAAAGTTCCGTAAGTTAATCATGGAGTATCGCGATAAGATGCTTGAGAAGTATGATACTCTTTGGAATTATGTATGGGTAGGCAATACGTCCCACATTCGTTTCCTCAAGACTATCGGTGCGGTATTCCATGAAGAGTACACACGAGATGGTCAATTTCAGTTATTTCCAATCACGAAAGGAGGATAACCATATGTGTTTGGCAGCCGCAATACCTATCGCTATATCTGGCCCTCAGGCTATc                                                                                                                                                                                                                                                                                    >  1:456990/1‑301 (MQ=255)`  
                           `aaaGTGCTGGTTCGTTACGAGCGACCAAGTGTGGCGACTTAGTGGAAAGGCTAAGCGAAAGTTCCGTAAGTTAATCATGGAGTATCGCGATAAGATGCTTGAGAAGTATGATACTCTTTGGAATTACGTATGGGTAGGCAATACGTCCCACATTCGTTTCCTCAAGACTATAGGTGCGGTATTCCATGAAGAGTACACACGAGATGGTCAATTTCAGTTATTTACAATCACGAAAGGAGGATAACCATATGTGTTGGGCAGCCGCAATACCTATCGCTATATCTGGCGCTCAGGCTATCAg                                                                                                                                                                                                                                                                                  <  1:746436/299‑1 (MQ=255)`  
                                                 `gACCGATTGTGGCGACTTAGTGGAAAGGCTATGCGAATGTTCCTTAAGTTACTCATGGAGTATCGCGATAAGATGCTTGAGAAGTATGATACTCTTTGGGATTACGTATGGGTAGGCAATACGTCCAACATTCGTTTCCTCAAGACTATCGGTGCGGTATTCCATGAAGAGTACACACGAGATGGTCAATTTCAGTTATTTACAATCACGAAAGGAGGATAACCATATTTGTTGGGCAGCCGCAATACCTATcgctatatctggcgctatatctggcgctcaggctatcagtggtcagaac                                                                                                                                                                                                                                                            <  1:807286‑M1/301‑50 (MQ=255)`  
                                                 `gACCAAGTGTGGCGACTTAGTGGAAAGGCTAAGCGAAAGTTCCATAAGTTAATCATGGAGTATCGCGATAAGATGCTTGAGAAGTATGATACTCTTTGTAATTACGGATGGGTAGGCAATACATCACACATGCGTTTCCTCAAGACTATCTGTGCGGTATTCAATGAAGATTACACACGAGATGATCAATTGCAGTTATTTACAATCAAGAACAAACAATAACCATATGTGGTGAGCAGCCGCAATACCTATcgctatatctggcgctaaatctggcgcaccagcctgcgatggtaagaag                                                                                                                                                                                                                                                            >  1:168614‑M1/1‑252 (MQ=255)`  
                                                   `agagacagggCGACTTAGTGGAAAGGCTAAGCGAAAGTTCCGTAAGTTAATCATGGAGTATCGCGATAAGATGCTTGAGAAGTATGATACTCTTTGGAATTACGTATGGGTAGGCAATACGTCCAACATTCGTTTCCTCAAGACTATCGGTGCGGTATTCCATGAAGAGTACACACGAGATGGTCAATTTCAGTTATTTACAATCACGAAAGGAGGATAACCATATGTGTTGGGCAGCCGCAATACCTATCGCTATATCTGGCGCTCAGGCTATAAGTGATCAGAACGCTCAGGCCAAAAt                                                                                                                                                                                                                                                          <  1:179142/293‑1 (MQ=255)`  
                                                                   `tgggctcggagatgtgtataagagacagaGTTAATCATGGAGTATTGCGATAAGATGCTTGAGAAGTATGATACTCTTTGGAATTACGTATGGGTAGGCAATACGTCCCACATTCGTTTCCTCAAGACTATCGGTGCGGTATTCCATGAAGAGTACACACGAGATGGTCAATTTCAGTTATTTACAATCACGAAAGGAGGATAACCATATGTGTTGGGCAGCCGCAATACCTATCGCTATATCTGGCGCTCAGGCTATCAGTGATCAGAACGCTCAGGCCAAAATGATTGCCGCTCAGAcc                                                                                                                                                                                                                                          <  1:696950/273‑1 (MQ=255)`  
                                                                    `gTGGAAAGGCTAAGCGAAAGTTCCGTAAGTTAATCATGGAGTATCGCGATAAGATGCTTGAGAAGTATGATACTCTTTGGAATTACGTATGGGTAGGCAATACGTCCCACATTCGTTTCCTCAAGACTATCGGTGCGGTATTCCATGAAGAGTACACACGAGATGGTCAATTTCAGTTATTTACAATCACGAAAGGAGGATAACCATATGTGTTGGGCAGCCGCAATACCTATCGCTATATCTGGCGATCAGGCTATCAGTGATCAGAACGCTCAGGCCAACATGAtctgtctcttataca                                                                                                                                                                                                                                         >  1:673719/1‑287 (MQ=255)`  
                                                                        `agagacagaGCGAAAGTTCCGTAAGTTAATCATGGAGTATCGCGATAAGATGCTTGAGAAGTATGATACTCTTTGGAATTACGTATGGGTAGGCAATACGTCCCACATTCGTTTCCTCAAGACTATCGGTGCGGTATTCCATGAAGAGTACACACGAGATGGTCAATTTCAGTTATTTACAATCACTAAAGGAGGATAACCATATGTGTTGGGCAGCCGCAATACCTATCGCTATATCTGGCGCTCAGGCTATCAGTGATCAGAACGCTCAGGCCAAAATGATTGCCGCTCAGACCgctgc                                                                                                                                                                                                                                     <  1:549937/293‑1 (MQ=255)`  
                                                                           `ggCTAAGCGAAAGTTCCGTAAGTTAATCATGGAGTATCGCGATAAGATGCTTGAGAAGTATGATACTCTTTGGAATTACGTATGGGTAGGCAATACGTCCCACATTCGTTTCCTCAAGACTATCGGTGCGGTATTCCATGAAGAGTACACACGAGATGGTCAATTTCAGTTATTTACAATCACGAAAGGAGGATAACCATATGTGTTTGGCAGCCGCAATACCTATCGCTATATCTGGCGCTCAGGCTATCAGTGATCAGAACGCTCAGGCCAAAATGATTGCCGCTCAGACCGCTGCTgg                                                                                                                                                                                                                                  >  1:761368/1‑301 (MQ=255)`  
                                                                               `aaGCGAAAGTTCCGTAAGTTAATCATGGAGTATCGCGATAAGATGCTTGAGAAGTATGATACTCTTTGGAATTACGTATGGGTAGGCAATACGTCCCACATTCGTTTCCTCAAGACTATCGGTGCGGTATTCCATGAAGAGTACACACGAGATGGTCAATTTCAGTTATTTACAATCACGAAAGGAGGATAACCATATGTGTTGGGCAGCCGCAATACCTATCGCTATATCTGGCGCTCAGGCTATCAGTGATCAGAACGCTCAGGCCAAAATGATTGCCGCTCAGACCGCTGCTGgtcgt                                                                                                                                                                                                                              >  1:44472/1‑301 (MQ=255)`  
                                                                                    `aaaGTTCCGTAAGTTAATCATGGAGTATCGCGATAAGATGCTTGAGAAGTATGATACTCTTTGGAATTACGTATGGGTAGGCAATACGTCCCACATTCGTTTCCTCAAGACTATCGGTGCGGTATTCCATGAAGAGTACACACGAGATGGTCAATTTCAGTTATTTACAATCACGAAAGGAGGATAACCATATGTGTTGGGCAGCCGCAATACCTATcgctatatctggcgctatatctggcgctcaggctatcagtggtcggaacgctcaggccaaaatgattgccgctcagaccgctgc                                                                                                                                                                                                                         <  1:187233‑M1/301‑85 (MQ=255)`  
                                                                                                `gTTAATCATGGAGTATCGCGATAAGATGCTTGAGAAGTATGATACTCTTTGGAATTACGTATGGGTAGGCAATACGTCCCACATTCGTTTCCTCAAGACTATCGGTGCGGTATTCCATGAAGAGTACACACGAGATGGTCAATTTCAGTTATTTACAATCACGAAAGGAGGATAACCATATGTGTTGGGCAGCCGCAATACCTATCGCTATATCTGGCGCTCAGGCTATCAGTGATCAGAACGCTCAGGCCAAAATGATTGCCGCTCAGACCGCTGCTGGTCGTCGTCAAGCTATGGAAAt                                                                                                                                                                                                             >  1:50076/1‑301 (MQ=255)`  
                                                                                                    `aTCATGGAGTATCGCGATAAGATGCTTGAGAAGTATGATACTCTTTGGAATTACGTATGGGTAGGCAATACGTCCCACATTCGTTTCCTCAAGACTATCGGTGCGGTATTCCATGAAGAGTACACACGAGATGGTCAATTTCAGTTATTTACAATCACGAAAGGAGGATAACCATATGTGTTGGGCAGCCGCAATACCTATCGCTATATCTGGCGCTCAGGCTATCAGTGATCAGAACGCTCAGGCCAAAATGATTGCCGCTCAGACCGCTGCTGGTCGTCGTCAAGCTATGGAAATcctt                                                                                                                                                                                                         >  1:399459/1‑298 (MQ=255)`  
                                                                                                       `ctGGAGTATCGCGATAAGATGCTTGAGACGTATGATACTCTTTTGAATTACGTATTGGTAGGCAATACGTCCCACATTCGTTTCCTCAAGACTATCGGTGCTGTATTCCATGAAGAGTACCCACGCGATGGTCAATTTCAGTTATTTACAATCACGAAAGGAGGATAACCATATGTGTTGGGCAGCCGCAATACCTATCGCTATATCTGGCGCTCAGGCTATCAGTTATCAGAACGCTCAGGCCAAAATGATTGCCGCTCAGACCGCTGCTGGTCGTCGTCAAGCTATGGAAATCATGAgg                                                                                                                                                                                                      <  1:785364/300‑1 (MQ=255)`  
                                                                                                       `aTGGAGTATCGCGATAAGATGCTTGAGAAGTATGATACTCTTTGGAATTACGTATGGGTAGGCAATACGTCCCACATTCGTTTCCTCAAGACTATCGGTGCGGTATTCCATGAAGAGTACACACGAGATGGTCAATTTCAGTTATTTACAATCACGAAAGGAGGATAACCATATGTGTTGGGCAGCCGCAATACCTATcgctatatctggcgctatatctggcgctcagtctatcagtggtcagaatgctcaggccaaaatgattgccgctcagaccgctgctggtcgtcgtcaagctatg                                                                                                                                                                                                      >  1:578935‑M1/1‑198 (MQ=255)`  
                                                                                                          `gAGTATCGCGATAAGATGCTTGAGAAGTATGATACTCTTTGTAATTACGTATGGGTAGGCAATACGTCCCACATTCGTTTCCTCAAGACTATCGGTGCGGTATTCCATGAAGAGTACACACGAGATGGTCAATTTCAGTTATTTACAATCACGAAAGGAGGATAACCATATGTGTTGGGCAGCCGCAATACCTATcgctatatctggcgctatatctggcgctcaggctatcagtggtcataacgctcaggccaaaatgattgccgctcagaccgctgctggtcgtcgtcaagctatggaa                                                                                                                                                                                                   <  1:451915‑M1/301‑107 (MQ=255)`  
                                                                                                              `aTCGCGATAAGATGCTTGAGAAGTATGATACTCTTTGGAATTACGTATGGGTAGGCAATACGTCCCACATTCGTTTCCTCAAGTCTATCGGTGCGGTATTCCATGAAGAGTACACACGAGATGGTCAATTTCAGTTATTTACAATCACGAAAGGAGGATAACCATATGTGTTGGGCAGCCGCAATACCTATCGCTATATCTGGCGCTCAGGCTATCAGTGATCAGAACGCTCAGGCCAAAATGATTGCCGCTCAGACCGCTGCTGGTCGTCGTCAAGCTATGGAAATCATGAGGCatgtct                                                                                                                                                                                               >  1:813727/1‑296 (MQ=255)`  
                                                                                                                 `gcgATAAGATGCTTGAGAAGTATGATACTCTTTGGAATTACGTATGGGTAGGCAATACGTCCCACATTCGTTTCCTCAAGACTATCGGTGCGGTATTCCATGAAGAGTACACACGAGATGGTCAATTTCAGTTATTTACAATCACGAAAGGAGGATAACCATATGTGTTGGGCAGCCGCAATACCTATCGCTATATCTGGCGCTCAGGCTATCAGTGGTCAGAACGCTCAGGCCAAAATGATTGCCGCTCAGACCGCTGCTGGTCGTCGTCAAGCTATGGAAATCATGAGGCAGACTAACa                                                                                                                                                                                            >  1:158322/1‑301 (MQ=255)`  
                                                                                                                  `cgATAAGATGCTTGAGAAGTATGATACTCTTTGGAATTACGTATGGGTAGGCAATACGTCCCACATTCGTTTCCTCAAGACTATCGGTGCGGTATTCCATGAAGAGTACACACGAGATGGTCAATTTCAGTTATTTACAATCACGAAAGGAGGATAACCATATGTGTTGGGCAGCCGCAATACCTATcgctatatctggcgctatatctggcgctcaggctatcagtggtcagaacgctcaggccaaaatgattgacgctcagaccgctgctggtcgtcgtcaagctatggaaatcatgag                                                                                                                                                                                           <  1:117945‑M1/301‑115 (MQ=255)`  
                                                                                                                    `aTAAGATGCTTGAGAAGTATGATACTCTTTGGAATTACGTATGGGTAGGCAATACGTCCCACATTCGTTTCCTCAAGACTATCGGTGCGGTATTCCATGAAGAGTACACACGAGATGGTCAATTTCAGTTATTTACAATCACGAAAGGAGGATAACCATATGTGTTTGGCAGCCGCAATACCTATCGCTATATCTGGCGCTCAGGCTATCAGTGATCAGAACGCTCAGGCCAAAATGATTGCCGCTCAGACCGCTGCTGGTCGTCGTCAAGCTATGGAAATCATGAGGCAGACGAACATcc                                                                                                                                                                                         >  1:521494/1‑301 (MQ=255)`  
                                                                                                                      `gggagatggggataagggaccgctgtttGGAATTACGTAGGGGTAGGCACTACGACCCACATTCGTTTCCTCAAGACTATCGGTGCGGTATTCCATGAAGAGTACACACGAGATGGTCAATTTCAGTTATTTACAATCACGAAAGGAGGATAACCATATGTGTTGGGCAGCCGCAATACCTATCGCTATATCTGGCGCTCAGGCTATCAGTGATCAGAACGCTCAGGCCAAAATGATTGCCGCTCAGACCGCGGCTGGTCGTAGTCAAGCTATGGAAATCATGAGGCAGACGAACATCCAg                                                                                                                                                                                       <  1:797081/276‑1 (MQ=255)`  
                                                                                                                                `ctcggagatgtgtataagagacagacgtatgggtaggcaatacgtcccaCATTCGTTTCCTCAAGACTATCGGTGCGGTATTCCATGAAGAGTACACACGAGATGGTCAATTTCAGTTATTTACAATCACGAAAGGAGGATAACCATATGTGTTGGGCAGCCGCAATACCTATcgctatatctggcgctatatctggcgctaaggctatcagtggtcagaacgctcaggccaaaatgattgccgctcagaccgctgctggtcgtcgtcaagctatggaaatcatgaggcagacgaacatcc                                                                                                                                                                             <  1:67763‑M1/277‑129 (MQ=255)`  
                                                                                                                                    `gTATGATACTCTTTGGAATTACGTATGGGTAGGCAATACGTCCCACATTCGTTTCCTCAAGACTATCTGTGCGGTATTCCATGAAGAGTACACACGAGATGGTCAATTTCAGTTATTTACAATCACGAAAGGAGGATAACCATATGTATTGGGCAGCCGCAATACCTATCGCTATATCTGGCGCTCAGGCTATCAGTGATCAGAACGCTCAGGCCAAAATGATTGCCGCCCAGACCGCTGCTGGTAGTCGTCAAGCTATGGAAATCATGAGGCAGACGAACAAccgtctcttataaacacc                                                                                                                                                                         >  1:40683/1‑285 (MQ=255)`  
                                                                                                                                    `gTATGATACTCTTTGGAATTACGTATGGGTAGGCAATACGTCCCACATTCGTTTCCTCAAGACTATCGGTGCGGTATTCCATGAAGAGTACACACGAGATGGTCAATTTCAGTTATTTACAATCACGAAAGGAGGATAACCATATGTGTTGGGCAGCCGCAATACCTATCGCTATATCTGGCGCTCAGGCTATCAGTGATCAGAACGCTCAGGCCAAAATGATTGCCGCTCAGACCGCTGCTGGTCGTCGTCAAGCTATGGAAATCATGAGGCAGACGAACATCCAGAATGCTGACCTATc                                                                                                                                                                         >  1:601211/1‑301 (MQ=255)`  
                                                                                                                                      `aTGATACTCTTTGGAATTACGTATGGGTAGGCAATACGTCCCACATTCGTTTCCTCAAGACTATCTGTGCGGTATTCCATGAAGAGTATACACGAGATGGTCAATTTCAGTTATTTACAATCACGAAAGGAGGATAACCATATGTGTTGGGCAGCCGCAATACCTATcgctatatctggcgctatatctggcgctcaggctatcagtggtcagaacgctcaggccaaaatgattgccgctcagaccgctgctggtcgtcgtcaagctatggaaatcatgaggcagacgaacatccagctgt                                                                                                                                                                       >  1:37716‑M1/1‑167 (MQ=255)`  
                                                                                                                                              `ctTTGGAATTACGTATGGGTAGGCAATACGTCCCACATTCGTTTCATCAAGACTATCGGTGCGGTATTCCATGAAGAGTACACACGAGATGGTCAATTTCAGTTATTTACAATCACGAAAGGAGGATAACCATATGTGTTGGGCAGCCGCAATACCTATcgctatatctggcgctatatctggcgctcaggctatcagtggtcagaacgctcaggccaaaatgattgcagctcagaccgctgctggtcgtcgtcaagctatggaaatcatgaggccgacgaacatccagactgctgaccta                                                                                                                                                               >  1:634653‑M1/1‑159 (MQ=255)`  
                                                                                                                                                         `cGTATGGGTAGGCAATACGTCCCACATTCGTTTCCTCAAGACTATCGGTGCGGTATTCCATGAAGAGTACACACGAGATGGTCAATTTCAGTTATTTACAATCACGAAAGGAGGATAACCATATGTGTTGGGCAGCCGCAATACCTATcgctatatctggcgctatatctggcgctcaggctatcagtgatcagaacgctcaggccaaaatgattgccgctcagaccgctgctggtcgtcgtcaagctatggaaatcatgaggcagacgaacatccagaatgctgacctatcgttgcaagc                                                                                                                                                    >  1:485050‑M1/1‑148 (MQ=255)`  
                                                                                                                                                                     `ccaTACGTCCCACATTCGTTTCCTCAAGACTATCGGTGCGGTATTCCATGAAGAGTACACACGAGATGGTCAATTTCAGTTATTTACAATCACGAAAGGAGGATAACCATATGTGTTGGGCAGCCGCAATACCTATCGATATATCTGGCGCTCAGGCTATCAGTGATCAGAACGCTCAGGCCAAAATGATTGCCGCTCAGACCGCTGCTGGTCGTCGTCAAGCTATGGAAATCATGAGGCAGACGAACATCCAGAATGCTGACCTATCGTTGCAAGCTCGAAGTAAACTTGAGGAAGCGTc                                                                                                                                        >  1:305721/3‑301 (MQ=255)`  
                                                                                                                                                                       `aTACGTCCCACATTCGTTTCCTCAAGACTATCGGTGCGGTATTCCATTAAGAGTACACACGAGATGGTCAATTTCAGTTATTTACAATCACGAAAGGAGGATAACCATATGTGTTGGGCAGCCGCAATACCTATcgctatatctggcgctatatctggcgctcaggctatcagtggtcagaacgctcaggccaaaatgattgccgcccagaccgctgctggtcgtcgtcaagctatggaaatcatgaggcagacgaacatccagaatgctgacctatcgttgcaagctcgaagtaaacttg                                                                                                                                      >  1:715390‑M1/1‑134 (MQ=255)`  
                                                                                                                                                                       `aTACGTCCCACATTCGTTTCCTCAAGACTATCGGTGCGGTATTCCATGAAGAGTACACACGAGATGGTCAATTTCAGTTATTTACAATCACGAAATGAGGATAACCATATGTGTTGGGCAGCCGCAATACCTATCGCTATATCTGGCGCTCAGGCTATCAGTGATCAGAACGCTCATGCCAAAATGATTGCCGCTCAGACCGCTGCTGGTCGTCGTCAAGCTATGGAAATCATGAGGCAGACGAACATCCAGAATGCTGACCTATCGTTGCAAGCTCGAAGTAAACTTGAGGAAGCGTccc                                                                                                                                      >  1:482034/1‑300 (MQ=255)`  
                                                                                                                                                                                        `tcggagatgtgtataagagacagaTTCCATGAAGAGTACACACGAGATGGTCAATTTCAGTTATTTACAATCACGAAAGGAGGATAACCATATGTGTTGGGCAGCCGCAATACCTATCGCTATATCTGGCGCTCAGGCTATCAGTGATCAGAACGCTCAGGCCAAAATGATTGCCGCTCAGACCGCTGCTGGTCGTCGTCAAGCTATGGAAATCATGAGGCAGACGAACATCCAGAATGCTGACCTATCGTTGCAAGCTCGAAGTAAACTTGAGGAAGCGTCCGCCGAGTTGACATCACAg                                                                                                                     <  1:350219/278‑1 (MQ=255)`  
                                                                                                                                                                                          `ccTCAAGACTATCGGTGCGGTATTCCATGAAGAGTACACACGAGATGGTCAATTTCAGTTATTTACAATCACGAAAGGAGGATAACCATATGTGTTGGGCAGCCGCAATACCTATCGCTATATCTGGCGCTCAGGCTGTCAGTGATCAGAACGCTCAGGCCAAAATGATTGCCGCTCAGACCGCTGCTGGTCGTCGTCAAGCTATGGAAATCATGAGGCAGACGAACATCCAGAATGCTGACCTATCGTTGCAAGCTCGAAGTAAACTTGAGGAAGCATCCGCCGAGTTGACCCCACAGaa                                                                                                                   >  1:329500/1‑301 (MQ=255)`  
                                                                                                                                                                                           `cTCAAGACTATCGGTGCGGTATTCCATGAAGAGTACACACGAGATGGTCAATTTCAGTTATTTACAATCACGAAAGGAGGATAACCATATGTGTTGGGCAGCCGCAATACCTATCGCTATATCTGGCGCTCAGGCTATCAGTGATCAGAACGCTCAGGCCAAAATGATTGCCGCTCAGACCGCTGCTGGTCGTCGTCAAGCTATGGAAATCATGAGGCAGATGAACATCCAGAATGCTGACCTATCGTTGCAAGCTCGAAGTAAACTTGAGGAAGCGTCCGCCTAGTTGACCCCACAGAAc                                                                                                                  >  1:34211/1‑301 (MQ=255)`  
                                                                                                                                                                                           `cTCAAGACTATCGGTGCGGTATTCCATGAAGAGTACACACGAGATGGTCAATTTCAGTTATTTACAATCACGAAAGGAGGATAACCATATGTGTTGGGCAGCCGCAATACCTATCGCTATATCTGGCGCTCAGGCTATCAGTGATCAGAACGCTCAGGCCAAAATGATTGCCGCTCAGACCGCTGCTGGTCGTCGTCAAGCTATGGAAATCATGAGGCAGACGAACATCCAGAATGCTGACCTATCGTTGCAAGCTCGAAGTAAACTTGAGGAAGCGTCCGCCGAGTTGACCCCACAGAAc                                                                                                                  >  1:323922/1‑301 (MQ=255)`  
                                                                                                                                                                                                 `aCTATTGGTGCGGTATTCCATGAAGAGTACACACGAGATGGTCAATTTCAGTTATTTACAATCACGAAAGGAGGATAACCATATGTGTTGGGCAGCCGCAATACCTATCGCTATATCTGGCGCTCAGGCTATCAGTGATCAGAACGCTCAGGCCAAAATGATTGCCGCTCAGACCGCTGCTGGTCGTCGTCAAGCTATGGAAATCATGAGGCAGACGAACATCCAGAATGCTGACCTATCGTTGCAAGCTCGAAGTAAACTTGAGGAAGCGTCCGCCGAGTTGACCACACAGAACATGCAg                                                                                                            <  1:361867/301‑1 (MQ=255)`  
                                                                                                                                                                                                  `atgtgtataagagacagcATGAAGAGTACACACGAGATGGTCAATTTCAGTTATTTACAATCACGAAAGGAGGATAACCATATGTGTTGGGCAGCCGCAATACCTATCGCTATATCTGGCGCTCAGGCTATCAGTGATCAGAACGCTCAGGCCAAAATGATTGCCGCTCAGACCGCTGCTGGTCGTCGTCAAGCTATGGAAATCATGAGGCAGACGAACATCCAGAATGCTGACCTATCGTTGCAAGCTCGAAGTAAACTTGAGGAAGCGTCCGCCGAGTTGACCACACAGAACATGCAGa                                                                                                           <  1:755258/284‑1 (MQ=255)`  
                                                                                                                                                                                                     `tCGGTGCGGTATTCCATGAAGAGTACACACGAGATGGTCAATTTCAGTTATTTACAATCACGAAAGGAGGATAACCATATGTGTTGGGCAGCCGCAATACCTATCGCTATATCTGGCGCTCAGGCTATCAGTGATCAGAACGCTCAGGCCAAAATGATTGCCGCTCAGACCGCTGCTGGTCGTCGTCAAGCTATGGAAATCATGAGGCAGACGAACATCCAGAATGCTGACCTATCGTTGCAAGCTCGAAGTAAACTTGAGGAAGCGTCCGCCGAGTTGACCCCACAGAACATGCAGAAgg                                                                                                        <  1:657055/301‑1 (MQ=255)`  
                                                                                                                                                                                                      `cGGTGTGGTATTCCATGAAGAGTACACACTAGATGGTCAATTTCAGTTATTTACAATCACGAAAGGAGGATAACCATATGTGTTGGGAAGCCGCAATACGTATCGCTATATCTGGCGCTCAGGCTATCAGTGGTCAGCACGCTCAGGCCAAAATGATTGCCGCTCAGACCGCTGCTGGTCGTCGTCAAGCTATGGAAATCATGAGGCAGACGAACATCCAGAATGCTGACCTATCGTTGCAAGCTCGAAGTAAACTTGAGGAAGCGTACGCCGAGTTGACCTCACAGAACATGCAGAAGGt                                                                                                       >  1:336211/1‑301 (MQ=255)`  
                                                                                                                                                                                                       `ggTTCGGTATTCCCTGAAGAGTACACCCGTGATGGTCAATTTCAGTTATTTACAATCACGAAAGGAGGATAACCATATGTGTTGGTCAGCCAAAATACCTATCGCTATATCTGGCGCTCAGGCTATCAGTGATCAGAACGCTCAGGCCAAAATGATTTCCGCTCAGACCGCTGCTGGTCGTCGTCAAGCTATGGAAATCATGAGGCAGACGAACATCCAGAATGCTGACCTATCGTTGCAAGCTCGAAGTAAACTTGAGGAAGCGTCCGCCGAGTTGACCCCACAGAACATGCAGAAGGTc                                                                                                      <  1:424330/301‑1 (MQ=255)`  
                                                                                                                                                                                                             `gTATTCCATGAAGAGTACACACGAGATGGTCAATTTCAGTTATTTACAATCACGAAAGGAGGATAACCATATGTGTTGGGCAGCCGCAATACCTATCGCTATATCTGGCGCTCAGGCTATCAGTGATCAGAACGCTCAGGCCAAAATGATTGCCGCTCAGACCGCTGCTGGTCGTCGTCAAGCTATGGAAATCATGAGGCAGACGAACATCCAGAATGCTGACCTATCGTTGCAAGCTAGAAGTAAACTTGAGGAAGCGTCCGCCGAGTTGACCCCACAGAACATGCAGAAGGTctgtctc                                                                                                >  1:267035/1‑295 (MQ=255)`  
                                                                                                                                                                                                             `gTATTCCATGAAGAGTACACACGAGATGGTCAATTTCAGTTATTTACAATCACGAAAGGAGGATAACCATATGTGTTGGGCAGCCGCAATACCTATCGCTATATCTGGCGCTCAGGCTATCAGTGATCAGAACGCTCAGGCCAAAATGAATGCCGCTCAGACCACTGCTGGTCGTCGTCAAGCTATGGAAATCATGAGGCAGACGAACATCCAGAATGCTGACCTATCGTTGCAAGCTCGAAGTAAACTTGAGGAAGCGTCCGCCGAGTTGACCCCACAGAACATGCAGAAGGTCCAAGCt                                                                                                <  1:274219/301‑1 (MQ=255)`  
                                                                                                                                                                                                                 `taagagacaggTACACACGAGATGGTCAATTTCAGTTATTTACAATCACGAAAGGAGGATAACCATATGTGTTGGGCAGCCGCAATACCTATCGCTATATCTGGCGCTCAGGCTATCAGTGATCAGAACGCTCATGCCAAAATGATTGCCGCTCAGACCGCTGCTGGTCGTCGTCAAGCTATGGAAATCATGAGGCAGACGAACATCCAGAATGCTGACCTATCGTTGCAAGCTCGAAGTAAACTTGAGGAAGCGTCCACCGAGTTGACCCCACAGAACATGCAGAAGGTCCAAGCTATTg                                                                                            <  1:620110/291‑1 (MQ=255)`  
                                                                                                                                                                                                                 `tCCATGAAGAGTACACACGAGATGGTCAATTTCAGTTATTTACAATCACGAAAGGAGGATAACCATATGTGTTGGGCAGCCGCAATACCTATCGCTATATCTGGCGCTCAGGCTATCAGTGATCAGAACGCTCAGGCCAAAATGATTGCCGCTCAGACCGCTGCTGGTCGTCGTCAAGCTATGGAAATCATGAGGCAGACGAACATCCAGAATGCTGACCTATCGTTGCAAGCTCGAAGTAAACTTGAGGAAGCGTCCGCCGAGTTGACCCCACAGAACATGCAGAAGGTCCAAGCTATTg                                                                                            >  1:384860/1‑301 (MQ=255)`  
                                                                                                                                                                                                                   `cATGAAGAGTACACACGAGATGGTCAATTTCAGTTATTTACAATCACGAAAGGAGGATAACCATATGTGTTGGGCAGCCGCAATACCTATcgctatatctggcgctatatctggcgctcaggctatcagtggtcagaacgctcaggccaaaatgattgccgctcagaccgctgctggtcgtcgtcaagctatggaaatcattaggcagacgaacatccagaatgctgacctatcgttgcaagctcgaagtaaacttgaggaagcgtccgccgagttgaccccacagaacatgcagaaggtc                                                                                          <  1:507115‑M1/301‑212 (MQ=255)`  
                                                                                                                                                                                                                    `aTGAAGAGTAAACAAGAGAGGGTCAAGTGCAGTGATTTACAAGCAAGAAAGGAGGAGAACCATAGGTGTTGGGCAGCAGCAATAACTATCGCTATATCCGGCGCTAAGGCTATCAGTGATAAGAACGCTGAGGACAAAATGAGTGCAGCTCAGACCGCTGCTGTTCGTCGTCAAGCTATGGAAATCATGAGGCAGACGAACATCCAGAATGCTGACCTATCGTTGCAAGCTCGAAGTAAACTTGAGGAAGCGTCCGCCGAGTTGACCCCACAGAACATGCAGAAGGTCCAAGCTATTGGGt                                                                                         <  1:130587/301‑1 (MQ=255)`  
                                                                                                                                                                                                                      `gtgtataagagacaggaTGGTCAATTTCAGTTATTTACAATCACGAAAGGAGGATAACCATATGTGTTGGGCAGCCGCAATACCTATCGCTATATCTGGCGCTCAGGCTATCAGTGATCAGAACGCTCAGGCCAAAATGATTGCCGCTCAGACCGCTGCTGGTCGTCGTCAAGCTATGGAAATCATGAGGCAGACGAACATCCAGAATGCTGACCTATCGTTGCAAGCTCGAAGTAAACTTGAGGAAGCGTCCGCCGAGTTGACCCCACAGAACATGCAGAAGGTCCAAGCTATTGGGTCt                                                                                       <  1:142319/286‑1 (MQ=255)`  
                                                                                                                                                                                                                      `gAAGAGTACACACGAGATAGTCAATTTCAGTTATTTACAATCACGAAAGGAGGATAACCATATGTGTTGGGCAGCCGCAATACCTATCGCTATATCTGGCGCTCAGGCTATCAGTGATCAGAACGCTCAGGCCAAAATGATTGCCGCTCAGACCGCTGCTGGTCGTCGTCAAGCTATGGAACTCATGAGGCAGACGAACATCAAGAATGCTGACCTATCCTTGCAAGCTCGAATTAAACTTGAGGAAGAGTCCGCCGCGTTGACCCCACAGAAAATCCAGAAGGTCCAAGCTACTTGGTCt                                                                                       >  1:206486/1‑301 (MQ=255)`  
                                                                                                                                                                                                                         `gtgtataagagacagggTCAATTTCAGTTATTTACAATCACGAAAGGAGGATAACCATATGTGTTGGGCAGCCGCAATACCTATCGCTATATCTGGCGCTCAGGCTATCAGTGATCAGAACGCTCAGGCCAAAATGATTGCCGCTCAGACCGCTGCTGGTCGTCGTCAAGCTATGGAAATCATGAGGCAGACGAACATCCAGAATGCTGACCTATCGTTGCAAGCTCGAAGTAAACTTGAGGAAGCGTCCGCCGAGTTGACCCCACAGAACATGCAGAAGGTCCAAGCTATTGGGTCTATc                                                                                    <  1:599265/286‑1 (MQ=255)`  
                                                                                                                                                                                                                         `gagTACACACGGGATGGTCAATTTCAGTTAATTACAATCACGAAAGGAGGATAACCATATGTGTTGGGCAGCCGCAATACCTATCGCTATATCTGGAGATCAGGCTATCAGTGATCAGAACGCTCAGGCCAAAATGATTGCCGCTCAGACCGCTGCTGGTCGTCGTCAAGCTATGGAAATCATGAGGCAGACGAACATCCAGAATGCTGACCTATCGTTGCAAGCTCGAAGTAAACTTGAGGAAGCGTACGCCGAGTTGACCCCACAGAACATGCAGAAGGTCCAAGCTATTGGGTCTATc                                                                                    <  1:751415/301‑1 (MQ=255)`  
                                                                                                                                                                                                                         `gagTACACACGAGATGGTCAATTTCAGTTATTTACAATCACGAAAGGAGGATAACCATATGTGTTGGGCAGCCGCAATACCTATCGCTATATCTGGCGCTCAGGCTATCAGTGATCAGAACGCTCAGGCCAAAATGATTGCCGCTCAGACCGCTGCTGGTCGTCGTCAAGCTATGGAAATCATGAGGCAGACGAACATCCAGAATGCTGACATATCGTTGCAAGCTCGAAGTAAACTTGAGGAAGCGTCCGCCGAGTTGACCCCACAGAAAATGCAGAAGGTCCAAGCTATTGGGTCTATc                                                                                    >  1:26056/1‑301 (MQ=255)`  
                                                                                                                                                                                                                           `gTACACACTAGATGGTCAATTTCAGTTATTTACAATCACGAAAGGAGGATAACAATATGTGTTGGGCAGCCGCAATACCTATcgctatatctggcgctatatctggcgctcaggctatcagtggtcagaacgctcaggccaaaatgattgccactcagaccgctgctggtcgtcgtcaagctatggaaatcatgaggcagacgaacatccagaatgctgacctatcgttgcaagctcgaagtaaacttgaggaagcgtccgccgagttgaccccacagaacatgcagaaggtccaagctat                                                                                  >  1:240076‑M1/1‑82 (MQ=255)`  
                                                                                                                                                                                                                           `gTACACACGAGATGGTCAATTTCAGTTATTTACAATCACGAAAGGATGATAACCATATGTGTTGGGCAGCCGCAATACCTATCGCTATATCTGGCGCTCAGGCTATCAGTGATCAGAACGCTCAGGCCAAAATGATTGCCGCTCAGACCGCTGCTGGTCGTCGTCAAGCTATGGAAATCATGAGGCAGACGAACATCCAGAATGCTGACCTATCGTTGCAAGCTCGAAGTAAACTTGAGGAAGCGTCCGCCGAGTTGACCCCACAGAACATGCAGAAGGTCCAAGCTATTGGGTCTATCCg                                                                                  <  1:727973/301‑1 (MQ=255)`  
                                                                                                                                                                                                                           `gTACACACGAGATGGTCAATTTCAGTTATTTACAATCACGAAAGGAGGATAACCATATGTGTTGGGCAGCCGCAATACCTATcgctatatctggcgctatatctggcgctcaggctatcagtgatcagaacgctcaggccaaaatgattgccgctcagaccgctgctggtcgtcgtcaagctatggaaatcatgaggcagacgaacatccagaatgctgacctatcgttgcaagctcgaagtaaacttgaggaagcgtccgccgagttgaccccacagaacatgcagaaggtcaaagctat                                                                                  >  1:25272‑M1/1‑82 (MQ=255)`  
                                                                                                                                                                                                                           `gTACACACGAGATGGTCAATTTCAGTTATTTACAATCACGAAAGGAGGATAACCATATGTGTTGGGCAGCCGCAATACCTATCGCTATATCTGGCGCTCAGGCTATCAGTGATCAGAACGCTCAGGCCAAAATGATTGCCGCTCAGACCGCTGCTGGTCGTCGTCAAGCTATGGAAATCATGAGGCAGACGAACATCCAGAATGCTGACCTATCGTTGCAAGCTCGAAGTAAACTTGAGGAAGCGTCCGCCGAGTTGACCCCACAGAACATGCAGAAGGTCCAAGCTATTGGGTCTATCCg                                                                                  <  1:610199/301‑1 (MQ=255)`  
                                                                                                                                                                                                                           `gTACACACGAGATGGTCAATTTCAGTTATTTACAATCACGAAAGGAGGATAACCATATGTGTTGGGCAGCCGCAATACCTATCGCTATATCTGGCGCTCAGGCTATCAGTGATAAGAACGCTCAGGCCAAAATGATTGCCGCTCAGACCGCTGCTGGTCGTCGTCAAGCTATGGAAATCATGAGGCAGACGAACATCCAGAATGCTGACCTATCGTTGCAAGCTCGAAGTAAACTTGAGGAAGCGTCCGCCGAGTTGACCCCACAGAACATGCAGAAGGTCCAAGCTATTGGGTCTATCCg                                                                                  <  1:474149/301‑1 (MQ=255)`  
                                                                                                                                                                                                                              `ctcggagatgtgtataagagacagaTTTACAATCACGAAAGGAGGATAACCATATGTGTTGGGCAGCCGCAATACCTATCGCTATATCTGGCGCTCAGGCTATCAGTGATCAGAACGCTCAGGCCAAAATGATTGCCGCTCAGACCGCTGCTGGTCGTCGTCAAGCTATGGAAATCATGAGGCAGACGAACATCCAGAATGCTGACCTATCGTTGCAAGCTCGAAGTAAACTTGAGGAAGCGTCCGCCGAGTTGACCTCACAGAACATGCAGAAGGTCCAAGCTATTGGGTCTATCCGAGc                                                                               <  1:773573/277‑1 (MQ=255)`  
                                                                                                                                                                                                                                `aacgAGATGGTCAATTTCAGTTATTTACAGTCACGAAAGGAGGATAACCATATGTGTTGGGCAGCCGCAATGCCTATcgctatatctggcgctatatctgtcgctcaggctatcagttatcagaacgctcaggccaaaatgattgctgctcagaacgctgctggacgtcgtcaagctatggaaatcatgaggcagacgaacatccagaatgctgacctatcgttgcaagctcgaagtaaacttgaggaagcgtccgccgagttgaccccacagaacatgcagaaggtccaagctattgggt                                                                             <  1:438049‑M1/300‑225 (MQ=255)`  
                                                                                                                                                                                                                                        `ggTCAATTTCAGTTATTTACAATCACGAAAGGAGGATAACCATATGTGTTGGGCAGCCGCAATACCTATCGCTATATCTGGCGCTCAGGCTATCAGTGATCAGAACGCTCAGGCCAAAATGATTGCCGCTCAGACCGCTGCTGGTCGTCGTCAAGCTATGGAAATCATGAGGCAGACGAACATCCAGAATGCTGACCTATCGTTGCAAGCTCGAAGTAAACTTGAGGAAGCGTCCGCCGAGTTGACCCCACAGAACATGCAGAAGGTCCAAGCTATTGGGTCTATCCGAGCGGCTATCGga                                                                     >  1:231797/1‑301 (MQ=255)`  
                                                                                                                                                                                                                                         `gTCAATTTCAGTTATTTACAATCACGAAAGGAGGATAACCATATGTGTTGGGCAGCCGCAATACCTAGCGCTATATCTGGCGCTCAGGCTATCAGTGATCAGAACGCTAAGGCCAAAATGATTGCCGATCAGACCGCTGCTGGTCGTCGTCAAGCTATGGAAATCATGAGGCAGACGAACATCCAGAATGCTGACCTATCGTTGCAAGCTCGAAGTAAACTTGAGGAAGCGTCCACCGAGTTGACCCCACAGAACATGCAGAAGGTCCAAGCTATTGGGTCTATCCGAGCGGCTATCGgag                                                                    <  1:86590/301‑1 (MQ=255)`  
                                                                                                                                                                                                                                         `gTCAAATTCAGTTATTTACAATCACGAAAGGAGGATAACCATATGTGTTGGGCAGCCGCAATACCTATCGCTATATCTGGCGCTCAGGCTATCAGTGATCAGAACGCTCAGGCCAAAATGATTGCCGCTCAGACCGCTGCTGGTCGTCGTCAAGCTATGGAAATCATGAGGCAGACGAACATCCAGAATGCTGACCTATCGTTGCAAGCTCGAAGTAAACTTGAGGAAGCGTCCGCCGAGTTGACCCCACAGAACATGCAGAAGGTCCAAGCTATTGGGTCTATCCGAGCGGCTATCGgag                                                                    <  1:703748/301‑1 (MQ=255)`  
                                                                                                                                                                                                                                           `taaTTTCAGTTATTTACAATCACGAAAGGAGGATAACCATATGTGTTGGGCAGCCGCAATACCTATCGCTATATCTGGCGCTCAGGCTATCAGTGATCAGAACGCTCAGGCCAAAATGATTGCCGCTCAGACCGCTGCTGGTCGTCGTCAAGCTATGGAAATCATGAGGCAGACGAACATCCAGAATGCTGACCTATCGTTGCAAGCTCGAAGTAAACTTGAGGAAGCGTCCGCCGAGTTGACCCCACAGAACATGCAGAAGGTCCAAGCTATTGGGTCTATCCGAGCGGCTATCGgagag                                                                  <  1:447081/300‑1 (MQ=255)`  
                                                                                                                                                                                                                                           `gagatgtgtataagagacagcacgaaaggaggataaccataTGTGTTGGGCAGCCGCAATACCTATcgctatatctggcgctatatctggcgctcaggctatcagtggtcagaacgctcaggccaaaatgattgcagctcagaccgctgctggtcgtagtcaagctatggaaatcatgaggcagacgaacatccagaatgctgacctatcgttgcaagctcgaagtaaacttgaggaagcgtccgccgagttgaccccacagaacatgcagaaggtccaagctattgggtctatccgagcg                                                                  <  1:356566‑M1/281‑236 (MQ=255)`  
                                                                                                                                                                                                                                           `cAATTTCAGTTATTTACAATCACGAAAGGAGGATAACCATATGTGTTGGGCAGCCGCAATACCTATCGCTATATCTGGCGCTCAGGCTATCAGTGATCAGAACGCTTAGGCCAAAATGATTGCCGCTCAGACCGCTGATGGTCGTCGTCAAGCTATGGAAATCATGAGGCAGACGAACATCCAGAATGCTGACCTATCGTTGCAAGCTCGAAGTAAACTTGAGGAAGCGTCCGCCGAGTTGACCCCACAGAACATGCAGAAGGTCCAAGCTATTGGGTCTATCCGAGCGGCTATCGgagag                                                                  <  1:666667/301‑1 (MQ=255)`  
                                                                                                                                                                                                                                           `aaaTTTCAGTTATTTACAATCACGAAAGGAGGATAACCATATGTGTTGGGCAGCCGCAATACCTATCGCTATATCTGGCGCTCAGGCTATCAGTGATCAGAACGCTCAGGCCAAAATGATTGCCGCTCAGACCGCTGCTGGTCGTCGTCAAGCTATGGAAATCATGAGGCAGACGAACATCCAGAATGCTGACCTATCGTTGCAAGCTCGAAGTAAACTTGAGGAAGCGTCCGCCGAGTTGACCCCACAGAACATGCAGAAGGTCCAAGCTATTGGGTCTATCCGAGCGGCTATCGgagag                                                                  <  1:290001/300‑1 (MQ=255)`  
                                                                                                                                                                                                                                             `aTTTCAGTTATTTACAATCACGAAAGGAGGATAACCATATGTGTTGGGCAGCCGCAATACCTATCGCTATATCTGGCGCTCAGGCTATCAGTGATCAGAACGCTCAGGCCAAAATGATTGCCGCTCAGACCGCTGCTGGTCGTCGTCAAGCTATGGATATCATGAGGCAGACGAAAATCCAGAATGCTGACCTATCGTTGCAAGCTCGAAGTAAACTTGAGGAAGCGTCCGCCGAGTTGACCCCACAGAACATGCAGAAGGTCCAAGCTATTGGGTCTATCCGAGCGGCTATCGGagagag                                                                >  1:68982/1‑301 (MQ=255)`  
                                                                                                                                                                                                                                              `tttCAGTTATTTACAATCACGAAAGGAGGATAACCATATGTGTTGGGCAGCCGCAATACCTATCGCTATATCTGGCGCTCAGGCTATCAGTGATCAGAACGCTCAGGCCAAAATGATTGCCGCTCAGACCGCTGCTGGTAGTCGTCAAGCTATGGAAATCATGAGGCAGACGAACATCCAGAATGCTGACCTATCGTTGCAAGCTCGAAGTAAACTTGAGGAAGCATCCGCCGAGTTGACCCCACAGAACATGCAGAAGGTCCAAGCTATTGGGTCTATACAAGCGGCTATCGgagaggga                                                               >  1:603204/1‑298 (MQ=255)`  
                                                                                                                                                                                                                                                   `tgggctaggagatgggtataagagacagccATATGTGTTGGGCAGCCGCAATACCTATCGCTATATCTGGCGCTCAGGCTATCAGTGATCAGAACGCTCAGGCCAAAATGATTGCCGCTCAGACCGCTGCTGGTCGTCGTCAAGCTATGGAAATCATGAGGCAGACGAACATCCAGAATGCTGACCTATCGTTGCAAGCTCGAAGTAAACTTGAGGAAGCGTCCGCCGAGTTGACCCCACAGAACATGCAGAAGGTCCAAGCTATTGGGTCTATCCGAGCGGCTATCGGAGAGAGTATGCt                                                          <  1:517339/273‑1 (MQ=255)`  
                                                                                                                                                                                                                                                   `taagagacagaTCATGAAAGGAGGATAACCATATGTGTTGGGCAGCCGCAATACCTATCGCTATATCTGGCGCTCAGGCTATCAGTGATCAGAACGCTCAGGCCAAAATGATTGCCGCTCAGACCGTTGCTGGTCGTCGTCAAGCTATGGAAATCATGAGGCAGACGAACATCCAGAATGCTGACCTATCGTTGCAAGCTCGAAGTAAACTTGAGGAAGCGTCCGCCGAGTTGACCCCACAGAACATGCAGAAGGTCCAAGCTATTGGGTATATCCGAGCGGCTATCGGAGAGAGTATGCt                                                          <  1:572178/291‑1 (MQ=255)`  
                                                                                                                                                                                                                                                   `gggctcggagatgtgtataagagacagaCCATATGTGTTGGGCAGCCGCAATACCTATCGCTATATCTGGCGCTCAGGCTATCAGTGATCAGAACGCTCAGGCCAAAATGATTGCCGCTCAGACCGCTGCTGGTCGTCGTCAAGCTATGGAAATCATGAGGCAGACGAACATCCATAATGCTGACCTATCGTTGCAAGCTCGAAGTAAACTTGAGGAAGCGTCCGCCGAGTTGACCCCACAGAACATGCAGAAGGTCCAAGCTATTGGGTCTATCCGAGCGGCTATCTGAGAGAGTATGCt                                                          <  1:319640/274‑1 (MQ=255)`  
                                                                                                                                                                                                                                                   `gTTACTTACAATCACGAAAGGAGGATAACCATATGTGTTGGGCAGCCGCAATACCTATCGCTATATCTGGCGCTCACGCTATCAGTGATCAGAACGCTCAGGCCAAAATGATTGCCGCTCAGACCGCTGCTGGTCGTCGTCAAGCTATGGAAATCATGAGGCAGACGAACCTCCAGAATGCTGAACTATCGTTGCAAGCTCGAAGTAAACTTGAGGAAGAATCCGCCGAGTTGACCCCACAGAACATGCAGAAGGTCCAAGCTATTGGGTCTATCTGAGCGGCTATCGGAGAGAGTATGCt                                                          <  1:556591/301‑1 (MQ=255)`  
                                                                                                                                                                                                                                                     `taagagacagcacgaaaggagGATAACCATATGTGTTGGGCAGCCGCAATACCTATcgctatatctggcgctatatctggcgctcaggctatcagtgatcagaacgctcaggccaaaatgattgccgctcagaccgctgctggtcgtcgtcaagctatggaaatcatgaggcagacgaacatccagaatgctgacctatcgttgcaagctcgaagtaaacttgaggaagcgtccgccgagttgaccccacagaacatgcagaaggtccaagctattgggtctatccgagcggctataggag                                                        <  1:16540‑M1/291‑246 (MQ=255)`  
                                                                                                                                                                                                                                                       `tttACAATCACGAAAGGAGGATAACCATATGTGTTGGGCAGCCGCAATACCTATcgctatatctggcgctatatctggcgctcaggctatcagtggtcagaacgctcaggccaaaatgattgccgctcagaccgctgctggtcgtcgtcaagctatggaaatcatgaggcagacgaacatccagaatgctgacctatcgttgcaagctcgaagtaaacttgaggaagcgtccgccgagttgacatcacagaacatgcagaaggtccaagctattgggtctatccgagcggctatcggagag                                                      <  1:41710‑M1/301‑248 (MQ=255)`  
                                                                                                                                                                                                                                                          `ggctcggagatgtgtataagagacagtgtgTTGGGCAGCCGCAATACCTATCGCTATATCTGGCGCTCAGGCTATCAGTGATCAGAACGCTCAGGCCAAAATGATTGCCGCTCAGACCGCTGCTGGTCGTCGTCAAGCTATGGAAATCATGAGGCAGACGAACATCCAGAATGCTGACCTATCGTTGCAAGCTCGAAGTAAACTTGAGGAAGCGTCCGCCGAGTTGACCCCACAGAACATGCAGAAGGTCCAAGCTATTGGGTCTATCCGAGCGGCTATAGGAGAGAGTATGCTTGAAGGt                                                   <  1:68209/275‑1 (MQ=255)`  
                                                                                                                                                                                                                                                           `agatgtgtataagagacagaccatatgtgttgggcagccGCAATACCTATcgctatatctggcgctatatctggcgctcaggctatcagtggtcagaacgctcaggccaaaatgattgccgctcagaccgctgctggtcgtcgtcaagctatggaaatcatgaggcagacgaacatccagaatgctgacctatcgttgcaagctcgaagtaaacttgaggaagcgtccgccgagttgacctcacagaacatgcagaaggtccaagctattgggtctatccgagcggctatcggagagagta                                                  <  1:427269‑M1/282‑252 (MQ=255)`  
                                                                                                                                                                                                                                                              `tCACGAAAGGAGGATAACCATATGTGTTGGGCAGCCACAATACCTATcgctatatctggcgctatatctggcgctcaggctatcagtggtcagaacgctcaggccaaaatgattgccgctcagaccgctgctggtcgtcgtcaagctatggaaatcatgaggcagacgaacatccagaatgctgacctatcgttgcaatctcgaagtaaacttgaggaagcgtccgccgagttgacctcacagaacatgcagaaggtccaagctattgggtctatccgagcggctatcggagagagtatgc                                               >  1:219453‑M1/1‑47 (MQ=255)`  
                                                                                                                                                                                                                                                               `cggagatgtgtataagagacaggtgttgggcagccgcaatacctatcgctatatctggcgctatatctggcgctcaggctatcagtggtcagaacgctcaggccaaaatgattgccgctcagaccgctgctggtcgtcgtcaagctatggaaatcatgaggcagacgaacatccagaatgctgacctatcgttgcaagctcgaagtaaacttgaggaagcgtccgcctagttgaccccacagaacatgcagaaggtccaagctattgggtctatccgagcggctatcggagagagtatgct                                              <  1:640689‑M1/279‑256 (MQ=255)`  
                                                                                                                                                                                                                                                               `cACGAAAGGAGGATAACCATATGTGTTGGGCAGCCGCAATACCTATCGCTATATCTGGCGCTCAGGCTATCAGTGATCAGAACGCTCAGGCCAAAATGATTGCCGCTCAGACCGCTGCTGGTCGTCGTCAAGCTATGGAAATCATGAGGCAGACGAACATCCAGAATGCTGACCTATCGTTGCAAGCTCGAAGTAAACTTGAGGAAGCGTCCGCCGAGTTGACCCCACAGAACATGCAGAAGGTCCAAGCTATTGGGTCTATCCGAGCGGCTATCGGAGAGAGTATGCTTGAAGGTTCCTc                                              <  1:792381/301‑1 (MQ=255)`  
                                                                                                                                                                                                                                                                  `gAAAGGAGGATAACCATATGTGTTGGGCAGCCGCAATACCTATCGCTATATCTGTCGCTCAGGCTATCAGTGATCAGAACGCTCAGGCCAAAATGATTGCCGCTCAGACCGCTGCTTGTCGTCGTCAAGCTATGGAAATCATGAGGCAGACGAACATCCATAATGCTGACCTATCGTTGCAAGCTCGAAGTAAACTTGAGGAAGCGTCCGCCGAGTTGACCCCACAGAACATGCAGAAGGTCCAAGCTATTGTGTCTATCCGAGCGGCTATCGGAGAGAGTATGCTTGAAGGTTCCTCAAt                                           <  1:531055/301‑1 (MQ=255)`  
                                                                                                                                                                                                                                                                         `ggaTAACCATATGTGTTGGGCAGCCGCAATACCTATcgctatatctggcgctatatctggcgctcaggctatcagtggtcagaacgctcaggccaaaatgattgccgctcagaccgctgctggtcgtcgtcaagctatggaaatcatgaggcagacgaacatccagaatgctgacctatcgttgcaagctcgaagtaaacttgaggaagcgtccgccgagttgacctcacagaacatgcagaaggtccaagctattgggtctatccgagcggctatcggagctgtctcttatacacatctc                                    >  1:146965‑M1/1‑36 (MQ=255)`  
                                                                                                                                                                                                                                                                             `aaCCATATGTGTTGGGCAGCCGCAATACCTATCGCTATATCTGGCGCTCAGGCTATCAGTGATCAGAACGCTCAGGCCAAAATGATTGCCGCTCAGACCGCTGCTGGTCGTCGTCAAGCTATGGAAATCATGAGGCAGACGAACATCCAGAATGCTGACCTATCGTTGCAAGCTCGAAGTAAACTTGAGGAAGCGTCCGCCGAGTTGAACCCACAGAACATGCAGAAGGTCCAAGCTATTGGGTCTATCCGAGCGGCTATCGGAGAGAGTATGCtctgtctcttatacacatctccgagcc                                >  1:819895/1‑275 (MQ=255)`  
                                                                                                                                                                                                                                                                               `ccATATGTGTTGGGCAGCCGCAATACCTATcgctatatctggcgctatatctggcgctcaggctatcagtggtcagaacgctcaggccaaaatgattgccgctcagaccgctgctggtcgtcgtcaagctatggaaatcatgaggcagacgaacatccagaatgctgacctatcgttgcaagctcgacgtaaacttgaggaagcgtccgccgagttgacctaacagaacatgcagaaggtccaagctattgggtctatccgagctgctatcggagagagtatgcttgaaggttcctcaatg                              >  1:64870‑M1/1‑30 (MQ=255)`  
                                                                                                                                                                                                                                                                                 `atatGTGTTGGGCAGCCGCAATACCTATCGCTATATCTGGCGCTCAGGCTATCAGTGATCAGAACGCTCAGGCCAAAATGATTTCCGCTCAGACCGCTGCTGGTCGTCGTCAAGCTATGGAAATCATGAGGCAGACGAACTTCCAGAATGCTTACCTATCGTTGCAAGCTCGAAGTAAACTTGAGGAAGCGTCCGCCGAGTTGACCCCACAGAACATGCAGAAGGTCCAAGCTATTGGGTCTATCCGAGCGGCTATCGGAGAGAGTATGCTTGAAGGTTCCTCAATGGACCGCATTAAGCg                            <  1:585526/301‑1 (MQ=255)`  
                                                                                                                                                                                                                                                                                  `aaagagatgggcagcCGCAATACCAATcgcaatatatggggctatatctagcgctaaggcgaacagtggtcagaacgctcaggccaaaaggattgcagctcagaccgctgctggtagtcgacaagcgatggaaatcatgaggcagacgaacaacaagaatactgacctatcgttgcaagctagaagtaaacttgaggaagcgtccgccgagatgaccccacagaaaatgcagaaggtccaagctattgggtctatccgagcggctatcggagagagtatgcttgaaggttcctcaatggac                           <  1:256982‑M1/294‑275 (MQ=255)`  
                                                                                                                                                                                                                                                                                     `gtgtTGGGCAGCCGCAATACCTATcgctatatctggcgctatatctggcgctcaggctatcagtgatcagaacgctcaggccaaaatgattgccgctcagaccgctgctggtcgtcgtcaagctatggaaatcatgaggcagacgaacatccagaatgctgacctatcgttgcaagctcgaagtaaacttgaggaagcgtccgccgagttgaccccacagaacatgcagaaggtccaagctattgggtctatccgagcggctatcggagagagtatgcttgaaggttcctcaatggaacgc                        >  1:561642‑M1/1‑24 (MQ=255)`  
                                                                                                                                                                                                                                                                                          `gggCAGCCGCAATACCTATCGCTATATCTGGCGCTCAGGCTATCAGTGATCAGAACGCTCAGGCCAAAATGATTGCCGCTCAGACCGCTGCTGGTCGTCGTCAAGCTATGGAAATCATGAGGCAGACGAACATCCAGAATGCTGACCTATCGTTGCAAGCTCGAAGTAAACTTGAGGAAGCGTCCGCCGAGTTGACCCCACAGAACATGCAGAAGGTCCAAGCTATTGGGTCTATCCGAGCGGCTATCGGAGAGAGTATGCTTGAAGGTTCCTCAATGGACCGCATTAAGCGAGTCACAGa                   <  1:280912/301‑1 (MQ=255)`  
                                                                                                                                                                                                                                                                                               `gCCGCAATACCTATCGCTATATCTGGCGCTCAGGCTATCAGTGATCAGAACGCTCAGGCCAAAATGATTGCCGCTCAGACCGCTGCTGGTCGTCGTCAAGCTATGGAAATCATGAGGCAGACGAACATCCAGAATGCTGACCTATCGTTGCAAGCTCGAAGTAAACTTGAGGAAGCGTCCGCCGAGTTGACCCCACAGAACATGCAGAAGGTCCAAGCTATTGGGTCTATCCGAGCGGCTATCGGAGAGAGTATGCTTGAAGGTTCCTCAATGGACCGCATTAAGCGAGTCACAGAAGGAc              <  1:108950/301‑1 (MQ=255)`  
                                                                                                                                                                                                                                                                                                `ccGCAATACCTATCGCTATATCTGGCGCTCAGGCTATCAGTGATAAGAACGCTCAGGCCAAAATGATTGCCGCTCAGACCGCTGCTGGTCGTCGTCAAGCTATGGAAATCATGAGGAAGACGAACATACAGAATGCTGACCTATCGTTGCAAGCTCGAAGTAAACTTGAGGAAGCGTCCGCCAAGTTGACCCCACAGAACATGCAGAAGGTCCAAGCCAGTGGGTCTATCCGAGCAGCTATCGGAGGGAGTATGCTTGAAGGTTCATCAATGGACCGCAGTAAGCGAGTCACAGAAGGACa             >  1:313608/1‑301 (MQ=255)`  
                                                                                                                                                                                                                                                                                                     `aTACCTATcgctatatctggcgctatatctggcgctcaggctatcagtggtcagaatgctcaggccaaaatgattgccgctcagaccgctgctggtcgtcgtcaagctatggaaatcatgaggcagacgaacatccagaatgctgacctatcgttgcaagctcgaagtaaacttgaggaagcgcccgccgagttgaccacacagaacatgcagaaggtccaagctattgggtgtatccgagcggctatcggatagagtatgcttgaaggttcatcaatggagcgcattaagcgagtcacag        >  1:246‑M1/1‑8 (MQ=255)`  
                                                                                                                                                                                                                                                                                                       `aCCTATCGCTATATCTGGCGCTCAGGCTATCAGTGGTCAGAACGCTCAGGCCAAAATGATTGCCGCTCAGACCGCTGCTGGTCGTCGTCAAGCTATGGAAATCATGAGGCAGACGAACATCCAGAATGCTTATCTATCGTTGCAAGCTAGAAGTAAACTTGAGGAAGTGTCCGCCGAGTGGACCTCACAGAACATGCAGAATGGCCAAGCGATTGGTTCTAGCCGACCGGCTATCGGAGAGAGTATGCTTGAAGGTTCCTCAATGGACCGCATTAAGCGAGTCACAGAAGGACAGTTCAtt      >  1:136804/1‑301 (MQ=255)`  
                                                                                                                                                                                                                                                                                                       `aCCTATCGCTATATCTGGCGCTCAGGCTATCAGTGATCAGAACGCTCAGTCCAAAATGATTGCCGCTCAGACCGCTGCTGGTCGTCGTCAAGCTATGGAAATCATGAGGCAGACGAACATCCAGAATGCTGACCTATCGTTGCAAGCTCGAAGTAAACTTGAGGAAGCGTCCGCCGAGTTGACCCCACAGAACATGCAGAAGGTCCAAGCTATTGGGTCTATCCGAGCGGCTATCGGAGAGAGTATGCTTGAAGGTTCCTCAATGGACCGCATTAAGCGAGTCACAGAAGGACAGTTCAtt      >  1:199302/1‑301 (MQ=255)`  
                                                                                                                                                                                                                                                                                                        `ccTATtgctatatctggcgctatatctggcgctcaggctatcagtggtcagaacgctcaggccaaaatgattgccgctcagaccgctgctggtcgtcgtcaagctatggaaatcatgaggcagacgaacatccagaatgctgacctatcgttgcaagctcgaagtaaacttgaggaagcgtccgccgagttgacctcacagaacatgcagaaggtccaagctattgggtctatccgagcggctatcggagagagtatgcttgaaggttcctcaatggacctgtctcttatacacatctccc     >  1:77944‑M1/1‑5 (MQ=255)`  
                                                                                                                                                                                                                                                                                                        `ccTATCGCTAGATCTGGCGCTCAGGCTATCAGTGATCAGAACGCTCAGGCCAAAATGATTGCCGCTCAGACCGCTGCTGGTCGTCGTCAAGCTATGGAAATCATGAGGCAGACGAACATCCAGAATGCTGACCTATCGTTGCAAGCTCGAAGTAAACTTGAGGAAGCATCCGCCGAGTTGACCCCACAGAACATGCAGAAGGTCCAAGCTATTGGGTCTATCCGAGCGGCTATCGGAGAGAGTATGCTTGAAGGTTCCTCAATGGACCGCATTAAGCGAGTCACAGAAGGACAGTTCATTc     <  1:191775/301‑1 (MQ=255)`  
                                                                                                                                                                                                                                                                                                           `ttcgctatatctggcgctatatctggcgctcaggctatcagtggtcagaacgctcaggccaaaatgattgccgctcagaccgctgctggtcgtcgtcaagctatggaaatcatgaggcagacgaacatccagaatgctgacctatcgttgcaagctcgaagtaaacttgaggaagcgtccgccgagttgacctcacagaacatgcagaaggtccaagctattgggtctatccgagcggctatcggagagagtatgcttgaaggttcctcaatggaccgcattaagcgagtcacagaaggac  <  1:365605‑M1/300‑300 (MQ=255)`  

                                                                                                                                                                                                                                                                                                            `|`                                                                                                                                                                                                                                                                                                             
`TCTAGCTATCGGTGGTAACTGCGGGGACCAGTGCTGGTTCGTTACGAGCGACCAAGTGTGGCGACTTAGTGGAAAGGCTAAGCGAAAGTTCCGTAAGTTAATCATGGAGTATCGCGATAAGATGCTTGAGAAGTATGATACTCTTTGGAATTACGTATGGGTAGGCAATACGTCCCACATTCGTTTCCTCAAGACTATCGGTGCGGTATTCCATGAAGAGTACACACGAGATGGTCAATTTCAGTTATTTACAATCACGAAAGGAGGATAACCATATGTGTTGGGCAGCCGCAATACCTATCGCTATATCTGGCGCTCAGGCTATCAGTGGTCAGAACGCTCAGGCCAAAATGATTGCCGCTCAGACCGCTGCTGGTCGTCGTCAAGCTATGGAAATCATGAGGCAGACGAACATCCAGAATGCTGACCTATCGTTGCAAGCTCGAAGTAAACTTGAGGAAGCGTCCGCCGAGTTGACCTCACAGAACATGCAGAAGGTCCAAGCTATTGGGTCTATCCGAGCGGCTATCGGAGAGAGTATGCTTGAAGGTTCCTCAATGGACCGCATTAAGCGAGTCACAGAAGGACAGTTCATTCGGG  >  NC_001604.1/27453‑28052`

  

Alignment Legend

Aligned base mismatch/match (shaded by quality score): `ATCG/ATCG` `< 3 ≤` `ATCG/ATCG` `< 11 ≤` `ATCG/ATCG` `< 25 ≤` `ATCG/ATCG` `< 36 ≤` `ATCG/ATCG` `< 38 ≤` `ATCG/ATCG`

Unaligned base: `atcg`    Masked matching base: `atcg`    Alignment gap: `‑`    Deleted base: `‑`

Reads not counted as support for junction

read\_name Not counted due to insufficient overlap past the breakpoint.

read\_name Not counted due to not crossing MOB target site duplication.
